# Current status of coping with insomnia among community-dwelling older adults with mild cognitive impairment and insomnia: a qualitative study

**DOI:** 10.3389/fpsyt.2026.1765635

**Published:** 2026-02-25

**Authors:** Yuhe Zhang, Junyu Zhao, Aijing Wu, Yu Zhang, Baoying Zhang, Yaming Zhang, Wanxin Wu, Wanxin Lu

**Affiliations:** School of Nursing, Fujian Medical University, Fuzhou, Fujian, China

**Keywords:** community, insomnia, mild cognitive impairment, older adults, qualitative study

## Abstract

**Background:**

The prevalence of insomnia is relatively high among community-dwelling older adults with mild cognitive impairment (MCI), and inappropriate coping strategies for insomnia are the primary cause of its persistence. This study aims to comprehensively understand the current status of insomnia coping strategies among older adults with MCI and insomnia in the community to provide a reference basis for the subsequent formulation of targeted insomnia treatment strategies.

**Methods:**

A descriptive qualitative study was conducted in the health education room of a community health service center using purposive sampling to select older adults for one-on-one semi-structured interviews. The inclusion criteria were: (1) age ≥ 60 years; (2) residing in the community for ≥ 6 months; (3) meeting the diagnostic criteria for MCI as outlined in the Diagnostic and Statistical Manual of Mental Disorders, Fifth Edition (DSM-5) and the diagnostic criteria for insomnia as outlined in the International Classification of Sleep Disorders, Third Edition (ICSD-3); and (4) basic communication and comprehension abilities. Traditional content analysis was used to organize and analyze the data.

**Results:**

A total of 17 participants were ultimately included, and data saturation was achieved. The current coping status of insomnia among older adults with MCI and insomnia in the community was categorized into three themes and ten subthemes: (1) coping attitudes (including negative coping, positive coping, and ambivalent coping); (2) coping styles (including insomnia symptom coping, active intervention coping, and cognitive impairment coping); and (3) coping needs (including treatment, knowledge, cognitive, and support needs).

**Conclusions:**

It is essential to implement non-pharmacological insomnia intervention methods in older adults with MCI and insomnia. It is recommended that primary healthcare institutions, such as community health service centers, pay attention to the insomnia issues of older adults with MCI, help them establish correct sleep cognition, and explore targeted, cognition-friendly, non-pharmacological insomnia intervention strategies, with a focus on providing sleep behavior intervention.

## 1 Introduction

With the acceleration of global population aging, the incidence of age-related neurodegenerative diseases continues to increase. Alzheimer’s disease has become a global public health issue, and mild cognitive impairment (MCI), at its preclinical stage, has attracted significant attention from many researchers. According to the Diagnostic and Statistical Manual of Mental Disorders, Fifth Edition (DSM-5) (1), MCI is defined as a clinical state characterized by progressive decline in one or more cognitive domains without impairing daily living abilities and does not meet the criteria for dementia diagnosis. A recent study indicated that the global prevalence of MCI among community-dwelling adults is 15.56%, highlighting that this condition has become a significant global public health issue (2). Furthermore, the prevalence of MCI is relatively high in the elderly population in China. Epidemiological surveys indicate that approximately 38.77 million individuals aged 60 years and above in China are affected by MCI, with a prevalence rate as high as 15.5% (3). It should be noted that approximately 10–15% of patients progress to dementia annually, with a conversion rate to dementia exceeding one-third within five years (4).

However, cognitive impairment in patients with MCI not only affects multiple cognitive domains such as memory, language, attention, executive function, visuospatial function, and social cognition, but may also impair non-cognitive domains such as mental behavior, mood, sleep, activities of daily living, and frailty (5). Studies have shown that the prevalence of sleep disorders among community-dwelling older adults with MCI ranges from 57.52% to 70%, which is significantly higher than that among older adults with normal cognitive function (6, 7). Insomnia is the most common type of sleep disorder and often presents as a noncognitive symptom of mild cognitive impairment (8), the prevalence of insomnia in patients with MCI is as high as 50% (9). According to the International Classification of Sleep Disorders, Third Edition (ICSD-3) (10), insomnia primarily manifests as difficulty falling asleep, difficulty in maintaining sleep, early awakening, reduced sleep quality, and decreased total sleep time, often accompanied by daytime functional impairments. Cognitive function in patients with MCI is closely associated with insomnia, which not only reduces sleep quality but also accelerates cognitive decline and psychiatric or behavioral impairments in patients with MCI. Many studies have found that the sleep characteristics of older adults with MCI exhibit significant abnormal changes, primarily related to insomnia, including reduced total sleep time, decreased sleep efficiency, prolonged sleep latency, and increased frequency and duration of awakenings (11, 12), These changes are closely associated with further cognitive decline (13–16). Furthermore, numerous studies have found that insufficient sleep may contribute to neurodegeneration through mechanisms such as beta-amyloid deposition, increased tau protein levels, inflammation, and impaired clearance of interstitial fluid from the cerebrospinal fluid into the interstitial spaces (17–19). Therefore, adequate and high-quality sleep is crucial to reduce cognitive decline. Currently, international evidence-based prevention guidelines for AD (20) have explicitly identified sleep disorders, such as insomnia, as modifiable risk factors for cognitive impairment and have incorporated sleep management into primary prevention, emphasizing the importance of sleep issues in the prevention and treatment of cognitive impairment.

Currently, the “3P” model of insomnia proposed by Spielman et al. (21) in 1986 is widely used to explain the causes of the onset, development, and maintenance of insomnia. This model conceptualizes the etiology of insomnia into three categories: predisposing, precipitating, and perpetuating factors. The presence of perpetuating factors is the primary reason for the persistence of insomnia symptoms. Among these, the inappropriate behavioral coping mechanisms developed by patients with insomnia to compensate for sleep deprivation are an important factor contributing to the persistence of insomnia (22). Studies have indicated that many individuals with insomnia often adopt various inappropriate behaviors to cope with sleep difficulties, such as going to bed early to compensate for perceived sleep loss, delaying morning rise times, or taking frequent daytime naps. During nighttime awakenings, they may either remain in bed in an attempt to force sleep or get up and engage in activities such as using smartphones or watching television to self-induce sleep (23). These behaviors not only fail to alleviate insomnia but may also contribute to its persistence. Several studies have elucidated the underlying mechanisms from different perspectives. For example, prolonged daytime napping can disrupt circadian rhythm and diminish nocturnal sleep drive, thereby hindering the improvement of insomnia (24–26). Going to bed early to extend sleep opportunities may actually increase nighttime awakenings, leading to more fragmented sleep and trapping individuals in a vicious cycle of excessive effort to sleep, which may also increase anxiety, further exacerbating the condition (27, 28). In summary, inappropriate coping behaviors can undermine sleep capacity, both physiologically and cognitively, thereby contributing to the chronicity of insomnia.

Although previous studies have confirmed that inappropriate coping strategies are a major factor in maintaining insomnia, most current research relies on patients’ self-reported sleep diaries to better understand their coping strategies. This approach often limits the understanding of sleep behavior to descriptions in these diaries, which are insufficiently in-depth and comprehensive. Few studies have employed qualitative research methods to thoroughly explore patients’ coping strategies, and even fewer have examined the unique coping strategies of individuals with insomnia who also have cognitive impairments, such as MCI. Therefore, this study aims to gain a deeper understanding of the coping strategies of community-dwelling older adults with MCI and insomnia through semi-structured interviews to provide a reference basis for the development of insomnia intervention and treatment methods tailored to this population.

## 2 Methods

### 2.1 Study design

The purpose of this study was to comprehensively understand the current status of insomnia coping among older adults with MCI and insomnia in the community; therefore, a descriptive qualitative research method was adopted. The descriptive qualitative research method was chosen because it is well suited for directly and systematically describing and exploring the experiences, perceptions, and behavioral patterns of a specific group of people regarding a phenomenon that is highly compatible with the research objectives. This method can capture the subjective experiences and specific practices of the patients themselves in coping with insomnia and carefully depict the types of coping strategies, implementation processes, perceived effects, and challenges, thus providing rich and detailed descriptive data for understanding insomnia coping in this group.

### 2.2 Participants and sampling strategies

Purposeful sampling was employed to select older adults diagnosed with MCI and insomnia in a specific community between March and April 2025 as study participants. The inclusion criteria were as follows: (1) age ≥ 60 years; (2) residing in the community for ≥ 6 months; (3) meeting the diagnostic criteria for MCI as outlined in the Diagnostic and Statistical Manual of Mental Disorders, Fifth Edition (DSM-5) (1) and the diagnostic criteria for insomnia as outlined in the International Classification of Sleep Disorders, Third Edition (ICSD-3) (10); and (4) basic communication and comprehension abilities. Exclusion criteria were: (1) language, hearing, or visual impairments; (2) cognitive impairments caused by other medications, such as antiepileptic drugs; (3) severe mental disorders, such as schizophrenia, or substance or alcohol addiction, who were unable to understand and cooperate with the interview; (4) other neurological disorders causing brain dysfunction or severe physical illnesses affecting daily life; (5) any other form of sleep disorder besides insomnia, such as sleep apnea or restless legs syndrome; and (6) Individuals who have experienced acute illness, trauma, or other significant life events in the past six months.

To ensure maximum sample diversity, various demographic variables (age, gender, educational attainment, etc.), and different severity levels of insomnia (assessed using the Insomnia Severity Index (ISI), where scores of 0–7 indicate no insomnia, 8–14 indicates mild insomnia, 15–21 indicates moderate insomnia, and 22–28 indicates severe insomnia (29)), we ensured the representativeness and typicality of the interview sample to obtain as comprehensive and rich data as possible.

### 2.3 Setting and context

This study was conducted in the health education room of a community health service center in Fuzhou City, Fujian Province, China, using Mandarin Chinese (a common language based on Beijing pronunciation). The interview outline was initially drafted based on a literature review and expert consultation, and then revised and finalized after conducting preliminary interviews with three elderly individuals. The final interview outline included the following seven questions: (1) Could you describe your current sleep schedule? For example, at what time do you usually go to bed at night? What time do you wake up in the morning? Do you have the habit of taking naps during the day? (2) How do you view your current insomnia issue? (3) Could you describe the specific manifestations of your current insomnia such as difficulty falling asleep, waking up frequently during the night, difficulty falling asleep after waking up, or waking up too early? Could you elaborate on these questions? (4) When insomnia symptoms occur (such as difficulty falling asleep, waking up frequently during the night, difficulty falling asleep after waking up, or waking up too early in the morning), what do you usually do? (5) Have you tried any treatment method to improve your insomnia? How effective have they been? (6) To address your current insomnia issues, what kind of assistance would you like for community healthcare providers and your close family members and friends to provide? (7) Is there anything else you would like to add?

### 2.4 Data collection

Offline face-to-face, one-on-one semi-structured interviews were used to collect data and were conducted by the first author herself (a master’s degree student in nursing, female) who had received training related to qualitative research. We contacted older adults in the community who met the criteria and established a good relationship of trust with the respondents by carrying out community health education activities before the start of the interviews, then explained the purpose and significance of the study in detail and formally started the interviews after obtaining their formal consent and signing the informed consent form. During the interviews, the recording device was placed in an undetectable position, the entire interview was recorded, and field notes were written, including facial expressions, body language, and important points or ideas of the interviewees. The sample size during the study followed the principle of information saturation, which means that the interviews were stopped when the information obtained began to recur and no new, important themes emerged. Seventeen participants were interviewed face-to-face in this study, and the participants participated without shedding.

### 2.5 Data analysis

The audio recordings were transcribed verbatim into text within 24 hours of the interviews, containing the content of the interviewer’s field notes. All transcripts were organized using NVivo qualitative data analysis software (QSR International, V.15, 2025). The interview data were analyzed using the traditional content analysis method (30), with the following steps: ① Read the transcribed text carefully and repeatedly until a comprehensive understanding is achieved; ② Disassemble the data and conduct a line-by-line analysis to identify significant statements, which are then coded; ③ code and categorize recurring and meaningful statements to generate themes; ④ Identify similarities among themes to form thematic groups; ⑤ Repeat this process iteratively until data saturation is reached, meaning no new themes or sub-themes emerge; ⑥ Validate the thematic structure by returning the results to the respondents for verification of content authenticity; ⑦ Finally, summarize the main content. After confirming the validity of all themes, the first author translated all original valid Chinese sentences, codes, categories, and themes into English. Two bilingual experts were invited to review the consistency between the Chinese and the English versions. Inconsistencies were discussed and revised to ensure accuracy and coherence.

### 2.6 Rigor

To ensure the reliability of the research, the following measures were adopted in this study: First, after transcribing the recordings into text, the interviewers repeatedly verified the content with the participants, recounting their understanding of the responses to the participants, thereby ensuring the accuracy of the recorded information through a member verification mechanism. Simultaneously, reflective journals were used throughout the research process to help researchers maintain self-reflection on the essence of the data and related phenomena. Furthermore, the team invited a qualitative research expert to serve as a reviewer, who reviewed the research process and interpreted the research findings to validate the reliability of the study. Finally, triangulation was systematically implemented at three levels: (1) Investigator triangulation: The entire coding and analysis process was independently conducted by two trained qualitative researchers, followed by iterative discussions and consensus meetings with the corresponding author to finalize themes. (2) Data source triangulation: We cross-verified information collected from participants with diverse demographic characteristics, comparing and corroborating participants’ statements with caregiver reports and community resident archives. (3) Methodological triangulation: The primary interview data were supplemented and contextualized with data from participant observation notes and a review of individual case profiles.

### 2.7 Ethical considerations

This study was approved by the Ethics Review Committee for Biomedical Research at Fujian Medical University (code number: 2025-171). All participants were fully informed of the purpose of this study, and we emphasized the voluntary and confidential nature of the study, allowing them to withdraw at any time. Additionally, the research data were strictly confidential, and de-identification measures were implemented during data processing, particularly for audio recordings, using coded identifiers to represent study cases, ensuring that participants’ personal information remained confidential.

## 3 Results

In this study, data saturation was achieved after interviewing 17 participants, a total of 17 participants were ultimately included (coded P1-P17). The participants’ age ranged from 60 to 86 years, with a mean age of 71.24 ± 8.04 (mean ± SD). Among them, 7 were male (41.18%) and 10 were female (58.82%). The basic characteristics of participants are shown in **Table 1**. After analyzing the study results, we extracted three themes and ten subthemes, as shown in **Table 2**.

**Table 1:** Basic characteristics of participants (*n* = 17).

No.	Age	Gender	Educational level	Marital status	Occupation	Severity of insomnia
P1	78	Female	Junior high school	Unmarried	Administrative staff	Severe
P2	73	Male	College	Married	Administrative staff	Medium
P3	70	Male	Junior high school	Married	Enterprise personnel	Mild
P4	77	Male	High School	Married	Laborer	Mild
P5	62	Female	High school	Widowed	Finance staff	Mild
P6	70	Female	Undergraduate	Married	Administrative staff	Moderate
P7	66	Male	College	Married	Laborer	Medium
P8	61	Female	Elementary school	Married	Laborer	Moderate
P9	86	Female	High school	Married	Laborer	Mild
P10	80	Female	Junior high school	Married	Laborer	Moderate
P11	77	Female	High school	Married	Laborer	Moderate
P12	67	Male	College	Married	Enterprise personnel	Mild
P13	60	Female	High School	Married	Administrative staff	Mild
P14	68	Male	Undergraduate	Married	Science and technology personnel	Moderate
P15	70	Male	High School	Married	Laborer	Moderate
P16	84	Female	Elementary school	Married	Laborer	Moderate
P17	62	Female	Junior high school	Married	Teacher	Heavy

**Table 2:** Themes and subthemes.

Themes	Subthemes
3.1 Coping attitudes	3.1.1 Negative coping 3.1.2 Positive coping 3.1.3 Ambivalent coping
3.2 Coping styles	3.2.1 Insomnia symptom coping 3.2.2 Active Intervention coping 3.2.3 Cognitive impairment coping
3.3 Coping Needs	3.3.1 Treatment needs 3.3.2 Knowledge needs 3.3.3 Cognitive Needs 3.3.4 Support needs

### 3.1 Coping attitudes

#### 3.1.1 Negative coping

Some older adults suffer from insomnia and have repeatedly sought ineffective medical treatment, thus being disappointed with insomnia treatment.

*P2: “There is no way to cure it. I also saw and did some treatment in the original provincial hospital. It seems that the effect is not too obvious, you can’t cure it, there is no hope.”*

Some older adults express helplessness about their current insomnia situation, feel indifferent, and do not want to take steps to improve their insomnia.

*P4: “I am very negative. I don’t care anyway. If I can sleep, I sleep; if I can’t sleep, I just lie there. I don’t care. There’s nothing I can do about it. It’s been like this for so many years. I don’t want to bother with it anymore.”*

Some older adults do not care about the problem of insomnia and have been forced to accept it, which is a common disease in the elderly.

*P12: “I don’t think we should place too much emphasis on insomnia, because it’s normal for older adults to have trouble sleeping. There’s nothing we can do but accept it. It’s a condition that comes with age, and there’s no way around it.”*

#### 3.1.2 Positive coping

Unlike older adults with negative attitudes towards insomnia, some are willing to take active measures to improve their insomnia.

*P14: “I also took the initiative to check WeChat to learn about some non-medicinal methods, such as certain devices, like electrical stimulation and massage devices. I actively sought out information about these.”*

It is worth pointing out that during the interview, one elderly person had a very optimistic attitude towards insomnia, believing that optimism and acceptance of the current insomnia situation, and treating it correctly with a normal mind, would be beneficial to the improvement of insomnia.

*P9: “I suffer from insomnia, but I don’t resent it. I accept it and deal with it properly. I don’t feel burdened by insomnia. Overall, I have a fairly positive attitude. If you deal with sleep positively, it will be good for you. I think it’s normal for older people to have trouble sleeping. If you take sleeping pills casually, I think it will be bad for your health in the future. You should treat it with a normal attitude.”*

Some older adults are eager to seek treatment for insomnia and hope that the problem of insomnia in older adults can be best addressed.

*P7: “Is there any way to improve the quality of sleep? I don’t know much about medical treatments, but I’m wondering if there’s any way to improve the quality of sleep, especially the problem of waking up in the middle of the night and not being able to fall back asleep. This is an urgent issue I want to resolve, as sometimes I can’t fall back asleep for several hours.”*

#### 3.1.3 Ambivalent coping

Some older adults have misconceptions about insomnia and often resort to compulsive behaviors, which make it even more difficult to fall asleep and lead to feelings of irritability.

*P6: “I can’t sleep no matter what I do. I try hard to fall asleep, but I can’t. This often makes it even harder to fall asleep, which is very annoying. I can’t sleep all night, which makes me feel very frustrated.”*

Some elderly people would like to improve their insomnia; they actively take measures but have no effect and fall into distress.

*P15: “I really want to improve my insomnia, but I don’t know what methods are effective. I’ve tried Chinese herbal medicine and other Chinese patent medicines, but nothing works. It’s very frustrating. I just wish I could sleep a little, even if it’s just a little.”*

### 3.2 Coping styles

#### 3.2.1 Insomnia symptom coping

##### 3.2.1.1 Difficulty in falling asleep coping

When it is difficult to fall asleep, most of the elderly choose to lie in bed and do a series of things that are not related to sleep, such as the use of television, cell phones, radios and other electronic devices to try to “hypnotize,” some older people like to watch TV, watch cell phones, and some older people because of the easy fatigue of the eyes, like to listen to cell phone novels, music, news, or listen to the radio and so on.

*P4: “I can’t sleep when I go to bed so early, so I watch TV until it’s almost time to go to sleep.”*

*P6: “I don’t know what I can do if I can’t sleep, so I’ll take my cell phone to watch it, and then I’ll sleep better if I’m tired.”*

*P8: “When I can’t sleep, I listen to audiobooks and novels. I put them on my bedside table and listen to them without looking at them, because I can’t sleep even when I’m lying down. With nothing else to do, I might as well turn on a novel and listen to it to pass the time.”*

*P13: “When I can’t sleep, I listen to music with the volume turned down low and place it next to my pillow.”*

*P14: “Listening to the news, national affairs, some people are seaking, giving speeches, and then sometimes I might fall asleep during the process.”*

*P15: “Sometimes when I can’t sleep, I listen to the radio on the bedside table.”*

Some older adults chose to try to hypnotize themselves by lying in bed and thinking about things to tire their brains.

*P11: “When I can’t sleep, my mind tends to dwell on things, exhausting my brain.”*

For religious reasons, some older adults choose to chant in bed to hypnotize themselves.

*P16: “I recited sutras by myself. When I couldn’t sleep, I would stand on my bed and chant ‘Amitabha Buddha.’ After chanting for a while, I would feel sleepy and want to yawn.”*

Some older adults go to bed early to help themselves become sleepy.

*P13: “I used to go to bed at 11 o’clock, but then it was even harder to fall asleep. I just lay there tossing and turning, unable to sleep. So I thought I’d go to bed early and see if I could fall asleep faster that way.”*

Some older adults do not take any measures and choose to stay in bed and wait until they fall asleep.

*P15: “Sometimes when I can’t sleep, I just lie there quietly with my eyes closed, waiting to fall asleep.”*

In contrast, some older adults chose to take active measures to accelerate falling asleep by getting up and doing relaxation exercises, such as massaging sleep acupoints, if they could not sleep.

*P9: “I won’t lie there, because if I lie in bed tossing and turning, my mind will start racing and I’ll think about all sorts of random things. In that case, I might as well get up and do some massage exercises. I feel that massaging these sleep acupoints will have a hypnotic effect.”*

##### 3.2.1.2 Difficulty in maintaining sleep coping

When experiencing symptoms such as frequent waking up during the night and difficulty falling asleep, most elderly people still choose to lie in bed and use electronic devices in an attempt to induce sleep.

*P2: “If I can’t sleep, I just lie there and look at my phone, because I don’t know how to fall asleep.”*

However, some older adults believe that looking at electronic devices upon waking up will make them feel more alert and thus find it more difficult to fall asleep, so they avoid using electronic devices when they wake up at night.

*P10: “When I wake up, I usually try not to look at electronic devices, because once I do, I won’t be able to sleep anymore and my mind will become alert.”*

Some older adults choose to get up and perform relaxing activities to help them sleep, eat food, or lie in bed, and try to fall asleep by reciting sutras, counting sheep, or thinking about things.

*P10: “When you wake up, don’t just lie in bed. The longer you lie in bed, the harder it will be to fall asleep, so do something else to relax, such as massage.”*

*P12: “Actually, I wasn’t hungry, I just couldn’t sleep and didn’t know what to do, so I got up and ate something.”*

*P11: “I also get up to recite sutras, begging Buddha to bless me with a quick sleep. Sometimes I count sheep, 1234, but I still can’t fall asleep.*”

*P4: “Think about things that happened during the day or in the past, and see if thinking hard will help you sleep better.”*

In addition, some older adults wake up at night and check their time, then simply lie quietly in bed waiting to fall asleep again, resulting in increased wakefulness time in bed.

*P1: “After waking up at night, I would check the time, but no matter what time it was, I never fell asleep. I just lay quietly in bed, not playing with my phone, and waited until dawn. “*

##### 3.2.1.3 Early waking coping

In the case of early awakening, most of the elderly would not get up immediately, but would choose to sleep for a while to compensate for the lack of sleep during the night.

*P5: “When I wake up early, I don’t get out of bed. Sometimes I just continue sleeping, lying there for a while longer, wanting to sleep for another hour or two.”*

Some older adults do not go on to sleep but choose to lie in bed and look at their cell phones to keep themselves awake so that they do not miss the time to make breakfast.

*P4: “Sometimes when I wake up early, I check my phone to wake myself up, because if I go back to sleep, I’m afraid I’ll oversleep and won’t have time to make breakfast. So I force myself not to go back to sleep and wait until 6 o’clock to get up.”*

##### 3.2.1.4 Daytime dysfunction coping

Some older adults have reported that daytime dysfunction due to lack of sleep at night is mainly characterized by increased daytime sleepiness. Some older adults actively cope with daytime sleepiness in the form of usual hobby activities, such as playing mahjong, sunbathing, and watching exciting TV dramas.

*P5: “I used to feel very sleepy in the afternoon, but if I took a nap in the afternoon, I couldn’t sleep at night. So I tried not to sleep in the afternoon, but sometimes I would still feel sleepy sitting on the sofa. What should I do? I took a measure: I tried to go out and play mahjong in the afternoon for two or three hours, and then come back. This way, the afternoon passed quickly, and I didn’t have to worry about feeling sleepy.”*

*P11: “When I don’t sleep well at night, I feel very tired during the day, and I can’t do as much housework. But I don’t dare to sleep either. Now, when I feel sleepy at noon, I stand on the balcony and soak up some sun. I feel that sunbathing is quite good for me. It helps me replenish calcium and makes me feel more energetic. Or sometimes I just sit in the living room and watch an exciting TV series.”*

However, some older adults cope with daytime sleepiness by increasing the time of daytime naps, which is an incorrect compensatory behavior for insomnia.

*P10: “If I don’t take a nap during the day, I feel very tired and my mind feels foggy. I just feel exhausted, so I usually sleep for several hours during the day. Sometimes I sleep until 3 or 4 in the afternoon, or even until 7 or 8.”*

#### 3.2.2 Active Intervention coping

##### 3.2.2.1 Pharmacotherapy

Some elderly people choose sleeping pills to treat insomnia, reflecting that sleeping pills are only effective for a short time and are dependent.

*P14: “If you take sleeping pills tonight, you will be able to sleep, but if you don’t take them afterwards, you won’t be able to sleep, and you will feel like you are dependent on them, so the effect is limited.”*

Some older adults resist the use of sleeping pills and choose Chinese herbal medicine, but the effect does not last.

*P7: “I tried some traditional Chinese medicine, and it worked pretty well at first, but the effects didn’t last.*

Some elderly people also choose mild Western medicines, such as glutamine, to treat their condition, but the results are unsatisfactory.

*P1: “I have also taken guanisul, but the doctor said to take it morning and night, and I didn’t feel it was effective.”*

##### 3.2.2.2 Physical therapy

Some elderly people use massage to improve their sleep, but the effect is not obvious.

*P11: “I massaged this point on my arm and another point behind my ear, but I didn’t feel any effect.”*

Some older adults use therapeutic instruments such as electromagnetic physiotherapy to help improve sleep.

*P13: “I went for some physical therapy, and it helped a lot. Otherwise, sometimes I couldn’t sleep at all at night, but now my sleep has improved a lot.”*

##### 3.2.2.3 Exercise therapy

Some older adults choose to improve their sleep through regular exercise such as walking and swimming. Some older adults believe that these exercises are effective in improving sleep, whereas others believe that they are not very helpful.

*P5: “After dinner, I go for a short walk in the lower part of the mountain, and when I return, I feel sleepy.”*

*P1: “If I go for a walk, I usually go around 4 p.m. before dinner and come back to eat. I don’t feel like it has much effect.”*

*P14: “Actually, it doesn’t work, and you can’t say that insomnia will be cured just by swimming.”*

Some older adults are also interested in traditional Chinese medicine for health maintenance and choose to improve their sleep through activities such as the Eight Brocades, Tai Chi, and finger exercises, but the results vary from person to person.

*P1: “I did the Eight Brocades exercise for about 10 minutes, but it didn’t help.”*

*P5: “I think Tai Chi helps me because after I do Tai Chi in the morning, or sometimes again at noon, I feel sleepy in the evening. However, I can’t do it in the evening because I’m too excited after exercising, which makes it hard for me to sleep.”*

*P2: “There’s a five-finger exercise on WeChat, right? I tried it, but it didn’t seem to work.*”

*P9: “Sometimes I tap my five fingers like this, which helps me fall asleep. Doing this seems to improve my sleep a little.”*

##### 3.2.2.4 Behavioral therapy

Some of the elderly adopted the doctor’s advice and chose to avoid napping and use hobbies during the day to fill up the time to improve their sleep, and achieved good results.

*P5: “The doctor told me to avoid taking naps during the day and to go out and have fun. I think this method is useful. Now, I play mahjong and walk my dog in the afternoon. By filling up my daytime schedule, I sleep much better at night, which has really helped my sleep.”*

Some older adults also achieved good results by changing their unhealthy sleep behaviors, such as “going to bed only when they felt sleepy,” “avoiding looking at cell phones before bedtime,” and “avoiding drinking strong tea in the afternoon”.

*P5: “The doctor advised me not to stay in bed all the time and to sleep only when I feel tired. I believe this approach is beneficial if I stick to it.”*

*P9: “There is everything on a mobile phone, and when you look at it, your mind reacts. Therefore, you really shouldn’t look at your mobile phone before going to bed at night. I generally don’t look at my mobile phone at night because it excites me and makes it even harder to fall asleep.”*

*P12: “I rarely drink tea in the afternoon now, but if I don’t drink tea, I won’t be able to sleep all night, so it does have some effect.”*

Some elderly people try to improve their insomnia by changing their sleeping position, but this has no effect.

*P1: “I sleep on my left side, my right side, and flat on my back all day long because I can’t sleep in any other position. The doctor told me to sleep on my side, but it didn’t help at all.”*

##### 3.2.2.5 Dietary interventions

Some older adults choose to improve their sleep by eating health-promoting foods, but the results are unclear.

*P13: “I have been consuming wild ginseng, nattokinase, and selenium for 15 years. They claim that these substances are beneficial for sleep, but I have not noticed any significant improvement.”*

Some older adults say that eating certain health-promoting stewed dishes, such as stewed apples and red dates, or stewed longans, can help alleviate insomnia to a certain extent.

*P17: “Now, my dietary therapy consists of stewing apples and longan and drinking the broth in the morning. I do this every two or three days, and I feel that it helps relieve my insomnia. It does have some effect.”*

Moreover, some older adults choose to drink alcohol before bedtime in order to help them fall asleep.

*P12: “The best way is to drink until you are drunk and then go straight to sleep. It is easy to fall asleep when you are drunk.”*

Some older adults choose to drink milk before bedtime, believing that it helps them to fall asleep.

*P11: “I drink milk before bedtime now, and I feel that it really helps me fall asleep. It makes it easier for me to fall asleep.”*

##### 3.2.2.6 Psychotherapy

Some older adults believe that insomnia is related to anxiety and tension, so they choose to engage in relaxing activities, such as meditation and quiet sitting, to help themselves relax and fall asleep.

*P1: “The doctor told me to meditate, but there seemed to be so many steps that I couldn’t remember them all, so I just did deep breathing, which still seemed to help calm me down a little.”*

*P5: “Now, I try to relax and have fun during the day, and at night, I try to relax and sleep. Before going to bed, I meditate to relax, try not to think about other things, and keep telling myself to relax. This way, I can sleep better after meditating.”*

##### 3.2.2.7 Music therapy

Some older adults believe that certain types of soothing music can help them to fall asleep.

*P3: “I usually listen to Chinese medicine lullabies, which are good for calming the mind and nourishing the* sp*irit. This kind of hypnotic music is slightly better, and I can usually sleep until around 5 o’clock.”*

#### 3.2.3 Cognitive impairment coping

Older adults with MCI and insomnia often have impaired cognitive function, which can affect insomnia-related treatment because of issues such as memory loss and decreased executive function. First, most elderly people say that their memory has deteriorated significantly, and that they need external prompts to remember things.

*P10: “I feel like I have a bit of dementia. I forget things easily and have a poor memory. I* sp*end the whole day looking for this and that, so now I take a small notebook with me when I go to see the doctor and ask him to write things down for me. That way, when I get home, I can look at what I wrote in my notebook and won’t forget what the doctor told me to do.”*

Some elderly people choose to practice calligraphy and writing. On the one hand, it helps them calm down and sleep better; on the other hand, it exercises their brains.

*P1: “The doctor told me that calming my mind would help me sleep better, so I* sp*end my days at home slowly writing. Writing is also a good way to exercise my brain, which has become quite forgetful lately. I write to pass the time, and it helps me calm my mind.”*

In addition, one elderly person reported a decline in executive function and chose to simplify the steps of insomnia treatment because she was unable to complete all of the steps.

*P1: “The doctor told me to do relaxation exercises, which involved closing my eyes, taking deep breaths, and thinking about something. There were five steps in total, but it was too difficult and there were too many steps, so I just did the deep breathing exercises.”*

### 3.3 Coping needs

#### 3.3.1 Treatment needs

Most older adults with MCI and insomnia are reluctant to use sleeping pills because of concerns about side effects, such as dependence and memory loss. They hope that doctors can provide nonpharmacological treatment options for insomnia.

*P14: “I don’t want to use sleeping pills because they are addictive, so I have been very distressed. Some of my friends have become addicted to eszopiclone and can’t stop taking it. They take more and more, and can’t sleep without it. Therefore, I hope you can provide me with a treatment plan that does not involve medication.”*

*P15: “I don’t want to take sleeping pills. Once I start taking them, I’ll have to keep taking them. It’s best not to give me any more sleeping pills because I’m afraid that taking too many will make me confused and forgetful.”*

Some older adults hope to receive treatment for insomnia using methods that are currently recognized by the medical community and have no side effects.

*P3: “Anyway, see if there are any medically approved methods available. I mainly want you to start treatment, preferably one that is not harmful to the body.”*

#### 3.3.2 Knowledge needs

The demand for knowledge regarding insomnia treatment among older adults with community-based MCI and insomnia mainly manifests itself in two areas: treatment guidance and disease awareness. Some older adults hope to receive professional guidance on improving their insomnia to help them improve their sleep quality.

*P2: “We hope the doctor can advise us on how to improve our sleep quality and fall asleep quickly.”*

*P5: “I am trying to adjust it myself now, but I feel like I don’t know how to do it on my own. I think I still need your professional help.”*

Some older adults want to improve their understanding of insomnia and are eager to learn about its causes and effective treatment.

*P2: “Now, can you find any other complementary therapies or exercise methods?”*

*P3: “I just want to know what caused the insomnia.”*

#### 3.3.3 Cognitive needs

Compared to older adults with normal cognitive function, older adults with MCI and insomnia have special cognitive needs for insomnia treatment due to cognitive decline. Some older adults want the treatment of insomnia to be easy to remember, for example with the help of manuals.

*P3: “I see there is a bulletin board next to us with many brochures. Could you print some materials for us to take home after the treatment? The doctors always ask us to do some things, but we always forget them.”*

Some older adults also hope that the treatment can be simplified to implement steps that are simple and easy to understand.

*P1: “I hope you can explain it clearly and concisely. Don’t use too many complicated medical terms at once, as we won’t understand them. For example, with relaxation therapy, tell me step by step how to do it, explaining each step simply so that I can understand it better.”*

Other older adults were concerned about memory loss and easy forgetfulness and hoped that the treatment for insomnia could be repeated and reinforced to achieve regular lectures.

*P7: “If you want to talk about sleep, you can talk more about it. Can you hold lectures regularly? If it’s only once, we may forget what they heard after the lecture.”*

#### 3.3.4 Support needs

The support needs of older adults with MCI and insomnia are mainly reflected in two aspects: family and community support.

First, since the treatment of insomnia has not yet attracted sufficient public attention, some elderly people say that their children do not know the effective methods for improving sleep. At the same time, their children are busy with work and rarely have time to pay attention to their sleep; therefore, they are less willing to seek treatment for insomnia.

*P1: “I just lay there quietly, unable to sleep. My children said that at my age, I should be able to fall asleep just by closing my eyes. They are very busy with work, so I can’t communicate with them that much.”*

*P11: “I also told them that I couldn’t sleep, and they said that this is common among the elderly and there is nothing that can be done about it.”*

In contrast, some older adults said that their family members were very supportive of their treatment for insomnia.

*P2: “They will support me in undergoing treatment. If I have time at home, I will come.”*

*P5: “My children are also very supportive of me now. They try their best to relax me and not bother me with their affairs, so I can just adjust myself.”*

Second, most older adults hope that community health service centers can provide relevant support services for insomnia treatment, such as promoting insomnia-related knowledge and holding lectures on insomnia treatment.

*P5: “You should encourage older adults not to sleep at home all day. Tell them to go out and get some exercise and try to sleep less during the day. You should also encourage them not to take sleeping pills indiscriminately and see if adjusting their behavior in this way can help improve their sleep.”*

*P6: “It would be best if our community could organize some lectures to discuss effective methods, help us gain some knowledge, and see if we can adjust our bad habits on our own.”*

## 4 Discussion

This study found that community MCI older adults with insomnia present a complex insomnia coping state with negative, positive, and ambivalent attitudes. This attitudinal differentiation is closely related to the lack of knowledge about insomnia treatment and insufficient knowledge about insomnia in older adults with MCI, which is similar to the results of Ting Ding’s previous study, which found that a lack of knowledge about insomnia is closely related to the patients’ poor attitudes when coping with insomnia, and the attitudes of inattention and negligence further lead to the patients’ failure to intervene actively in the problem of insomnia, which then leads to a decline in their ability to cope with insomnia (31). In addition, a qualitative study by Zhou et al. found that MCI patients generally lacked correct perception of disease symptoms, and most believed that the appearance of the corresponding symptoms was caused by normal aging (32), suggesting that compared with older adults with normal cognitive function, older adults with MCI tend to have a lower ability to perceive disease. At present, research on chronic diseases in the elderly predominantly emphasizes targeted interventions for diagnosed common conditions, such as hypertension and diabetes. In contrast, insomnia has received relatively limited attention in the field of geriatric health, resulting in insufficient public education regarding insomnia awareness among older adults. Consequently, many community-dwelling older adults with MCI and insomnia fail to acquire relevant knowledge in a timely manner, leading to an inadequate understanding of insomnia, often causing them to underestimate their multifaceted impact on health, adopt a passive attitude toward management, and develop a negative and ambivalent perception of the condition. However, there is a close association between insomnia and cognitive decline, and many studies have shown that insomnia is a high risk factor for further cognitive decline in patients with MCI (13–15), and if older adults with MCI negatively cope with insomnia due to insufficient knowledge of insomnia, it may accelerate the further decline of cognitive function and increase the risk of transition to dementia. It should be noted that the American College of Physicians (33), American Academy of Sleep Medicine (34), European Sleep Society (35), and British Association of Psychopharmacology (36) have pointed out that cognitive behavioral therapy for insomnia (CBT-I) is preferred as the first choice of non-pharmacological therapy for insomnia in adult insomnia patients, especially in older adults. Furthermore, the Chinese Sleep Research Society (37) also pointed out that non-pharmacological treatment should be preferred for elderly patients with insomnia, with a particular emphasis on CBT-I. However, because of the relatively limited methods of diagnosis and treatment of insomnia and the lack of professional implementers in primary healthcare institutions, such as community health service centers in China, non-pharmacological treatments, such as CBT-I, have not been widely used, and traditional sedative-hypnotic and traditional Chinese medicine treatments are still dominant (38). Moreover, many older adults in the community suffer from potential inappropriate medication (PIM) due to issues such as frailty and multimorbidity (39), and the use of hypnotic agents is often associated with a high incidence of adverse reactions and concerns regarding drug dependence (40), leading to treatment discontinuation and suboptimal outcomes. These factors collectively diminish older adults’ willingness to seek medical care, resulting in a generally passive and ambivalent attitude toward managing insomnia. It is well known that for the elderly in the community, the primary medical institutions represented by community health service centers are often the most convenient choice for medical consultation, and previous studies have shown that the training of primary health care doctors in CBT-I components can bring clinically meaningful and continuous improvement of insomnia symptoms for patients in the community (41). Therefore, in the future, community health centers should actively organize relevant medical personnel to conduct cognitive behavioral therapy training for insomnia, strengthen the publicity of non-pharmacological treatment of insomnia represented by CBT-I, and break the single mode of only using medication, so as to provide increasingly lasting non-pharmacological treatment of insomnia in elderly people with insomnia who are suffering from MCI in the community, and then improve their willingness to be treated and change their attitude of coping with insomnia. Meanwhile, relevant medical staff of community health centers should also actively carry out insomnia knowledge popularization activities for older adults with MCI and their families, such as carrying out group lectures and distributing insomnia treatment manuals, which can raise the importance of insomnia and correct cognitive bias so as to encourage them to change their attitudes and actively cope with insomnia. Furthermore, community medical personnel can use digital resources such as the Internet to publicize insomnia treatment by combining online and offline methods, such as setting up WeChat groups to facilitate communication and regularly pushing popular science articles and short videos to expand publicity channels to improve the convenience of popularizing science.

In addition, this study showed that most older adults cope poorly with insomnia. First, regarding coping with insomnia symptoms, most older adults tend to adopt a series of wrong behaviors, which tend to further contribute to the persistence of their insomnia. Previous studies have found that patients with insomnia, due to insufficient nighttime sleep as well as the lack of correct cognition of insomnia, which is often accompanied by the daytime compensatory rest phenomenon, often take excessive sleep, such as daytime frequent and prolonged naps and other undesirable behaviors, which may reduce the nighttime sleep drive, thus further disrupting the circadian rhythm and leading to the persistence of insomnia (25, 42). Therefore, the relevant medical personnel of community health service centers should pay attention to the symptomatic coping of older adults with MCI and insomnia and guide them to improve their attention to the behavioral habits of sleep, thus motivating them to correct their wrong behaviors and develop a correct way of coping with insomnia. Second, regarding active intervention for insomnia, older adults with MCI and insomnia indicated that behavioral therapy is the most effective way to avoid daytime naps, refraining from drinking strong tea in the afternoon, reducing mobile phone use before sleep, and going to bed only when feeling sleepy significantly improved their sleep quality. This observation aligns closely with the therapeutic principles of CBT-I, which mainly consist of sleep hygiene education, stimulus control therapy, sleep restriction therapy, relaxation therapy, and cognitive therapy etc. (34). As a multicomponent intervention, CBT-I is designed to rectify patients’ maladaptive beliefs about insomnia and dysfunctional sleep-related behaviors through a series of cognitive restructuring and behavioral modification techniques, thereby helping them develop appropriate coping strategies for insomnia. This highlights the importance of community healthcare professionals in prioritizing the management of sleep-related behaviors in older adults with MCI and insomnia. This can be achieved by conducting regular assessments via interviews to identify the presence of poor sleep habits, followed by the implementation of a range of behavioral interventions for insomnia, such as sleep hygiene education, stimulus control therapy, and sleep restriction therapy, which can promptly address and correct these inappropriate sleep behaviors. Finally, it is well noted that older adults with MCI and insomnia often face additional cognitive challenges when managing sleep disturbances due to cognitive decline, which complicates insomnia treatment in this population. Consistent with previous research findings, these individuals exhibit reduced learning and memory capacities compared to their previous abilities, making it difficult for them to acquire and master conventional insomnia interventions. It is necessary to construct a scientific, feasible, and applicable sleep intervention program for the cognitive characteristics of older adults (43); therefore, subsequent interventions should consider the cognitive characteristics of older adults with MCI and adopt cognitive-friendly approaches in the treatment of insomnia, in order to reduce cognitive load and help older adults with MCI improve cognitive memory ability, which can enhance the feasibility of the treatment and make them more cooperative with the treatment. This can be achieved by increasing reminders through the use of social chat platforms online or paper reminder slips offline to strengthen the memory of older adults with MCI, and at the same time simplifying the steps of behavioral interventions so that they are easy to understand, which can help reduce the difficulty of implementation for older adults with MCI.

The results also demonstrated that community-dwelling older adults with MCI and insomnia have multiple needs for insomnia management. These needs reflect expectations for coping with sleep disturbances and provide guidance for the development of tailored insomnia interventions suited to this population in future research. First, treatment needs focused on the use of non-pharmacological, side-effect-free alternative therapies, consistent with the findings of a previous qualitative study exploring the experiences of patients with chronic insomnia, which showed that insomnia patients disliked sleeping pills and preferred other gentle, non-pharmacological alternatives such as Traditional Chinese Medicine (TCM), Acupuncture, Acupressure, or dietary changes (44). Meanwhile, a previous study investigating insomnia patients’ views on insomnia behavioral treatment in primary care also showed that most insomnia patients tend to learn behavioral skills to improve their sleep first, and prefer non-pharmacological behavioral treatments to pharmacological treatments (45), suggesting that community health service centers should consider non-pharmacological behavioral treatments, such as CBT-I, to minimize insomnia medications in their treatment choices of insomnia in elderly patients, th MCI and reduce the impact of adverse drug reactions on the cognition and other aspects of health of elderly people with MCI, and motivate them to actively treat insomnia from the perspective of correcting their poor sleep behaviors, in order to delay the decline of cognitive function. Second, knowledge demand manifests as older adults’ desire to obtain professional insomnia improvement guidance and to increase insomnia-related knowledge. Previous studies have found that sleep hygiene education can allow patients to learn more about the knowledge and principles of sleep, effectively improve the cognition of normal sleep, and help them understand the persistence factors that lead to insomnia, so as to enhance their self-confidence and motivation in the treatment (46, 47). This highlights the importance of community health service centers proactively providing older adults with insomnia with accessible guidance on sleep improvement knowledge, such as sleep hygiene education, as well as regularly organizing health promotion activities within the community to raise awareness about insomnia treatment. These efforts can enhance illness awareness among older adults with MCI, enabling them to recognize the detrimental effects of insomnia, and thereby manage insomnia appropriately under professional guidance. A key finding of this study is that the special needs of older adults with MCI for insomnia treatment are cognitive needs, which require insomnia treatment methods that are easy to remember, simple, and repeatable. This suggests that conventional non-pharmacological treatments, such as verbal explanations by doctors, have limited effects on older adults with MCI. A previous study by Zhang Ruifen et al. indicated that conventional non-pharmacological interventions for patients with MCI primarily relied on unimodal cognitive training, which often fails to stimulate patients’ initiative and engagement. In contrast, multimodal nursing interventions that integrate verbal, visual, and auditory stimuli can engage multiple sensory channels such as vision, touch, and hearing. This approach facilitates a more comprehensive understanding and retention of information, while also offering flexibility and interactivity, thereby enhancing patient acceptance and adherence (48), This suggests that multimodal information delivery methods could be considered to reduce cognitive load in older adults with MCI. For example, treatment manuals can be designed with text and images for easy memorization, or animated demonstrations of the implementation steps can be created with voiceovers. Finally, demand for support is manifested in the expectations of family care and community services. Some older adults reflected that their family members did not support them in treating insomnia, which may be due to the lack of family members’ knowledge of insomnia and their lack of concern for older adults with MCI. Family care is the closest social support system, which can provide patients with realistic care, companionship, and emotional communication, and good family functioning can improve patients’ sleep quality (49). Furthermore, most studies have pointed out that the support and understanding of family members can help alleviate the symptoms of insomnia (50–52). Therefore, community health service centers should emphasize the role of family support in the treatment of insomnia and consider involving family members to help them correctly understand insomnia, thereby guiding them to support and encourage older adults with MCI to actively accept treatment. At the same time, family members can also play a supervisory role to ensure that interventions are implemented on a regular basis. In addition, the elderly with MCI also reflected a lack of support services related to insomnia treatment in community health service centers, such as insufficient popularization of insomnia and insufficient publicity of treatment, leading to a lack of knowledge related to insomnia prevention and treatment. Previous research by Wang et al. pointed out that there is insufficient knowledge of sleep disorders among older adults in the community in China and that community health workers should follow the physiological-psychosocial-social medical model to educate older adults and their caregivers about sleep health-related knowledge, such as conducting community lectures and door-to-door knowledge dissemination (53). Therefore, community health service centers should consider leveraging the existing chronic disease management frameworks to integrate sleep management for older adults with MCI and insomnia into essential community services. By consolidating community resources and regularly organizing health education activities such as insomnia awareness lectures, corresponding sleep support services can be provided. These efforts would further establish a stepped home-community medical support system, thereby maximizing therapeutic support for this vulnerable population.

## 5 Limitations and recommendations for future research

While this study explored the current situation of insomnia among older adults with MCI and insomnia in the community, it is important to recognize that there are some limitations. The study was conducted in only one community health center in Fuzhou City, Fujian Province, which may limit the generalizability of the findings to scenarios such as other communities or rural healthcare institutions. Future studies should be replicated in different geographic areas to ensure broader applicability of the findings. In addition, the small sample size of this interview may not fully reflect the diversity of the current status of insomnia coping among older adults with mild cognitive impairment and insomnia in the community. Future studies should expand the sample size to include participants of different ages, socioeconomic backgrounds, and cultural backgrounds to enhance the persuasiveness of the findings.

Moreover, because the coping styles of insomnia are affected by coping attitudes, interviewing only patients may cause too much bias owing to the inclusion of subjective personal emotions. Future studies can further interview doctors in community health service centers, and interview the wrong coping styles of insomnia patients who come to the clinic from the doctors’ perspective, in order to establish a patient-doctor dyadic perspective to obtain more comprehensive insights into the coping status of insomnia among older adults with MCI and insomnia.

It is worth noting that although this study emphasized the importance of developing correct insomnia coping attitudes and styles in older adults with MCI and insomnia, insomnia interventions that are appropriate for the cognitive level of older adults with MCI still need to be further explored in subsequent studies. Future studies should attempt to construct cognitively adapted, non-pharmacological insomnia intervention protocols for older adults with MCI, in order to more effectively help older adults with MCI to improve their insomnia, which will in turn have a positive impact on cognitive function.

Finally, this study only interviewed older adults with MCI comorbid with insomnia, and the insomnia coping status of patients with other comorbid insomnia has not yet been investigated, which may have different coping styles and needs due to the different effects of the disease. In the future, we will continue to conduct qualitative research to explore the current insomnia coping status of the insomnia group with different disease comorbidities to provide targeted guidance for the treatment of insomnia in this group of people.

## 6 Conclusion

In this study, we explored the insomnia coping status of older adults with MCI through semi-structured interviews, and found that older adults with MCI had more negative attitudes toward insomnia, more incorrect ways of coping with insomnia, and more diverse needs for coping with insomnia. This finding suggests that nonpharmacological insomnia interventions for older adults with MCI and insomnia are of great value. Therefore, it is suggested that primary medical institutions, such as community health centers, should pay attention to the insomnia problem of older adults with MCI and formulate targeted and cognitively friendly non-pharmacological intervention strategies, strengthen professional training on non-pharmacological treatment of insomnia for relevant medical personnel in the primary community, and improve the ability of early identification of insomnia and behavioral interventions for older adults with MCI in the community. At the same time, they should carry out targeted sleep hygiene education as early as possible to help older adults with MCI establish correct sleep hygiene cognition. In the future, we will focus on providing behavioral interventions to improve insomnia by correcting erroneous sleep behavioral habits from the perspective of eliminating factors that maintain insomnia.

## Data Availability

The raw data supporting the conclusions of this article will be made available by the authors, without undue reservation.
